# Recreation for Middle Age

**Published:** 1893-05-06

**Authors:** 


### The Hospital

AN INSTITUTIONAL JOURNAL OF

**Science, Medicine, Nursing, and Philanthropy.**

*For Hospitals, Asylums, Infirmaries, Dispensaries, Medical Practitioners, Students, Nurses, and the Charitable Public.*

VOL. XIV.—No. 345.] MAY 6, 1893.

### RECREATION FOR MIDDLE AGE.

THERE are two ways of making the load of life easier and more tolerable—the one is to diminish its weight, the other to increase the strength of the carrier's shoulders and to raise his courage. An experienced man has learnt that the world of humanity has a certain load to carry, a load laid upon it by destiny, which all the restiveness and ill-temper in the world will not enable it to get rid of. A brave man does not usually wish to have his load lightened; rather he thinks he might carry a little more, and so relieve others who are less capable than himself.

The true business of life is to do as much work as possible, of the highest quality, and for the longest period. But in order to do this the thinking mind must be constantly used. The thinking mind puts us upon the right track. The man who thinks evolves, gradually, for himself a scheme of life whereby he gets the most and the best possible out of himself. Such a scheme of life always includes, as a chief constituent of its several parts, "recreation." Recreation is not the pleasure of the idler, but the refreshment of the toiler. The idler does not know, and cannot know, anything about recreation. The toiler makes it a part of the business of his life.

Mr. William Odell, F.R.C.S., has boiled down the opinions of at least forty-five eminent persons, living and dead, upon recreations, and given us the essence in a capital pamphlet of less than forty-five pages. The opinions include those of Sir James Paget and other eminent medical men; the Rev. Dr. Hornby, Provost of Eton, and other distinguished school masters; a bishop, a military officer, a naval officer, a lady gymnast, Lord Bacon, Charles Kingsley, and many other persons of weight and authority indisputable. Now none of those persons could have had anything to gain by expressing opinions about "recreation": none of them are or have been professional recreationists. Their testimony must have been delivered out of mere love, and in most, perhaps in every one of the cases, it is the testimony of experience. Is such testimony worth a little thought, hurrying and impatient reader? Undoubtedly it is.

Here is a series of facts, given on the authority of Sir William Savory, ex-President of the Royal College of Surgeons. Says Sir William: "The necessity of exercise to the preservation of health is allowed by everyone. . . . Take for instance the effect of muscular exercise on respiration. When at rest a man draws in 480 cubic inches of air per minute; if he walks at the rate of four miles an hour this quantity is increased fivefold, amounting to 2,400 cubic inches in the same time. Or to put it in another way: it was found by experiment that a man at rest inspired per hour 27 cubic feet of air. This represents the absorption of 416 8 grains of oxygen, and the exhalation of 603 grains of carbonic acid, equal to 164 grains of carbon. But during exercise he inspired in the same period 64 9 cubic feet of air, representing the absorption of 1,829·6 grains of oxygen and the exhalation of 2,501 grains of carbonic acid, equal to 682 grains of carbon." In other words, a man during active exercise in the open air takes in nearly five times as much life-giving oxygen and gets rid of nearly five times as much of death-dealing carbonic acid or waste matter as a man at rest. No amplification can make a fact like this more striking. The man who does not understand and act upon it in its bare simplicity has not got the mental capacity to understand and act upon it at all, even though a hundred physiological lecturers were to rise from the dead to proclaim it day and night in his foolish ears.

Walking exercise is but one form of recreation. This is what the Hon. and Rev. E. Lyttleton, Head Master of Haileybury College, says of our noble cricket: "It is a mercy that some ingredient is still left in school life which teaches inevitably and safely true self-reliance. The young Englishman of our day learns a little of a great many different subjects, but, excepting at cricket, self-reliance is not one of them. His intellectual path is smoothed by every conceivable form of help; every Greek play he reads is boiled down for him; his moral life is protected by unceasing vigilance; there is great danger lest the true object of public school training, self-dependance, be ignored in the prevailing anxiety to keep boys out of mischief. But when a boy is commanding a side at cricket he must, willy nilly, show if he has the making of a man in him or not."

Of the forty-five eminent persons quoted by Mr. Odell, every one is a sober enthusiast for recreation; every one insists that recreation is absolutely indispensable. The present writer is convinced that large numbers of the business and literary class destroy their working powers, ruin their health, and make middle and old age miserable by nothing else but the reluctance to take recreation, or the mental incapacity to choose a suitable form of recreation for themselves, and to stick to it. The subject in its entirety is much too wide for discussion in a newspaper article; but we are anxious to pin attention down to two particulars—first, the absolute necessity for recreation in some form; and, secondly, the necessity for recreation out of doors for all classes of men and women who are doing the brain work of the world. Nobody would believe, who has not tried it, the difference which a single hour's walking exercise every evening after dinner can make to a man in two or three months. The effect produced is like a return of youth.

Mere walking exercise, although it is invaluable, hardly fulfils the idea of perfect recreation. Sir James Paget says "good active recreations" ought to include "uncertainties, wonders, and opportunities for the exercise of skill in something different from the regular work." The present writer is always longing for cricket in the summer, and football or hockey in the winter and spring. But he cannot find a man anywhere above forty years of age who will agree with him. Why should the literary man, the doctor, and the stockbroker or the merchant not play cricket after forty-five? What is to become of his dinner hour, is it asked? If a better luncheon were taken at mid-day, and a lighter dinner at six in the evening, there is no reason whatever why a man of forty-five, and up to sixty-five or seventy should not be in the cricket-field at half past seven and play briskly until nine or half-past. An hour and a half at cricket after a light dinner would make middle-aged men so young that they would not know themselves. Writers would write twice as brilliantly, and business men would be cleverer and keener by half. As it is the average middle-aged Englishman of the professional and business classes grows fatter, wheezier, more pompous, and more dull and uninteresting every year of his life. To get a laugh out of him is impossible; to crack a joke at his expense is to commit the unpardonable sin. "Poor old porpoise," as somebody has called him. His innocent pleasures have vanished with his youth, and "he has nothing now left to live for but his respectability: his solemn respectability, and his money-bags."

The contrast between the youthful Englishman and his middle-aged parent is sometimes startling. The former is all life and fun; the latter is a moving mountain of ponderosity and fat. It is all for want of outdoor exercise and recreation. Twenty-five years ago the solemn father of to-day was the fun-loving son of a middle-aged father. If anybody had then shown him in a prophetic mirror the figure he would cut at the end of a quarter of a century he would have committed suicide in sheer vexation and disgust. But all this rotundity, wheeziness, irritability of temper, incapacity for work and general disgust with life and all things in it can be cured, cured easily, and cured for ever; and the cure for the vast majority of cases is one or two hours daily exercise and recreation in the open air.

